# Evaluation of Two Rapid Antigenic Tests for the Detection of SARS-CoV-2 in Nasopharyngeal Swabs

**DOI:** 10.3390/jcm10132774

**Published:** 2021-06-24

**Authors:** Ysaline Seynaeve, Justine Heylen, Corentin Fontaine, François Maclot, Cécile Meex, Anh Nguyet Diep, Anne-Françoise Donneau, Marie-Pierre Hayette, Julie Descy

**Affiliations:** 1 Department of Clinical Microbiology, Center for Interdisciplinary Research on Medicines (CIRM), University of Liège, 4000 Liège, Belgium; jheylen@student.uliege.be (J.H.); c.fontaine@chuliege.be (C.F.); francois.maclot@chuliege.be (F.M.); c.meex@chuliege.be (C.M.); mphayette@chuliege.be (M.-P.H.); julie.descy@chuliege.be (J.D.); 2 Biostatistics Unit, Department of Public Health, University of Liège, 4000 Liège, Belgium; anhnguyet.diep@uliege.be (A.N.D.); afdonneau@uliege.be (A.-F.D.)

**Keywords:** rapid diagnostic test, antigen detection, SARS-CoV-2, COVID-19

## Abstract

(1) Background: In the current context of the COVID-19 crisis, there is a need for fast, easy-to-use, and sensitive diagnostic tools in addition to molecular methods. We have therefore decided to evaluate the performance of newly available antigen detection kits in “real-life” laboratory conditions. (2) Methods: The sensitivity and specificity of two rapid diagnostic tests (RDT)—the COVID-19 Ag Respi-Strip from Coris Bioconcept, Belgium (CoRDT), and the coronavirus antigen rapid test cassette from Healgen Scientific, LLC, USA (HeRDT)—were evaluated on 193 nasopharyngeal samples using RT-PCR as the gold standard. (3) Results: The sensitivity obtained for HeRDT was 88% for all collected samples and 91.1% for samples with Ct ≤ 31. For the CoRDT test, the sensitivity obtained was 62% for all collected samples and 68.9% for samples with Ct ≤ 31. (4) Conclusions: Despite the excellent specificity obtained for both kits, the poor sensitivity of the CoRDT did not allow for its use in the rapid diagnosis of COVID-19. HeRDT satisfied the World Health Organization’s performance criteria for rapid antigen detection tests. Its high sensitivity, quick response, and ease of use allowed for the implementation of HeRDT at the laboratory of the University Hospital of Liège.

## 1. Introduction

In December 2019, a new virus belonging to the *Betacoronavirus* genus from the *Coronaviridae* family appeared in the city of Wuhan in China. It was identified as the agent responsible for a severe respiratory syndrome, hence its name: SARS-CoV-2 for Severe Acute Respiratory Syndrome Coronavirus. The virus is responsible for coronavirus disease 19 (COVID-19), so named because it appeared in 2019. Other betacoronaviruses involved in human respiratory syndromes include SARS-CoV-1—the agent of the Severe Acute Respiratory Syndrome discovered in 2002—and MERS-CoV, responsible for the Middle East Respiratory Syndrome in 2012 [1,2]. Within a few months, SARS-CoV-2 spread rapidly, leading to the World Health Organization’s (WHO) announcement of a COVID-19 pandemic on 11 March 2020 [3]. As of 3 June 2021, 171.3 million people have been infected and 3.7 million people have died worldwide [4]. This pandemic has caused microbiology labs to develop diagnostic methods based on antigen and RNA detection. The WHO defined a reverse transcription polymerase chain reaction (RT-PCR) assay on respiratory specimens as the reference method for the detection of SARS-CoV-2 [5]. This coronavirus is an enveloped virus with a positive-sense, single-stranded RNA genome of ~30 kb. Its genome encodes a minimum of 29 proteins. The ORF1a and ORF1b genes encode non-structural accessory proteins (nsps), while the S, M, E, and N genes encode structural proteins—namely, spike (S), membrane (M), envelope (E), and nucleocapsid (N) proteins [6,7]. These genes are of particular interest for the diagnosis of COVID-19 by RT-PCR.

The real-time RT-PCR assay provides a semi-quantitative estimation of viral concentration expressed by the cycle threshold (Ct), which represents the number of amplification cycles required to detect a fluorescence signal above the threshold. The cycle threshold is correlated to the viral load and contagiousness [8,9]. Despite the very high specificity and sensitivity of molecular techniques, RT-PCR is time-consuming and requires specific laboratory equipment and experienced technical staff [10,11]. These limitations—combined with the shortage of PCR reagents and disposables—have led laboratories to investigate alternatives. In September 2020, the WHO reported the possible use of antigenic tests as a promising complement to RT-PCR. The WHO described rapid diagnostic tests (RDT) with a sensitivity ≥80% and a specificity ≥97% compared to the reference RT-PCR method that can be used when molecular tests are not available or when rapid screening is needed [8]. Confronted with the second wave of COVID-19, laboratories were under pressure to perform RT-PCR for the detection of SARS-CoV-2. The turnaround time was longer than the acceptable range of 24 h, which resulted in delays to diagnosis that compromised patients and epidemic management [12]. In addition, the emergency department of our hospital (CHU, Liège, Belgium) needed an early diagnosis of SARS-CoV-2 in order to manage patient hospitalization in COVID or non-COVID care units. Many companies have developed RDTs for the diagnosis of COVID-19 as point-of-care testing methods. Despite the high sensitivity announced by the manufacturers, the performance of these tests is varied and often lower than expected; therefore, their validation in real conditions is important [13,14,15,16,17,18]. A validation of two RDT kits for the detection of SARS-CoV-2 compared to RT-PCR was conducted at the microbiology laboratory of CHU Liège. The two kits are both membrane-based immunochromatographic assays that detect SARS-CoV-2 nucleocapsid proteins: (1) the coronavirus antigen rapid test cassette (Healgen Scientific, LLC, Houston, TX, USA), named hereafter HeRDT, and (2) the COVID-19 Ag Respi-Strip (Coris Bioconcept, Gembloux, Belgium), referred to as CoRDT.

## 2. Materials and Methods

### 2.1. Study Design

The diagnostic accuracy of HeRDT and CoRDT compared to RT-PCR (defined as the gold standard) was determined in a two-phase validation. The first phase—with nasopharyngeal (NP) swabs selected by convenience—aimed to verify that the minimum expected performance was achieved based on the recommendation of minimum sensitivities and specificities described by the WHO—i.e., a sensitivity ≥80% and a specificity ≥97% [8]. The second phase carried out on randomized NP samples aimed to validate the RDT in real diagnostic conditions. 

### 2.2. Population and Study Period

Validation was conducted between 22 October 2020 and 11 November 2020 at CHU Liège, a tertiary hospital with 1038 beds in Belgium. We included NP-flocked swabs placed in 3 mL of transport media (Vacuette Virus Stabilization Tube (VST); Greiner Bio-One, Kremsmünster, Austria) containing phosphate-buffered saline. The samples were kept between 2 and 8 °C and tested within 24 h for both RT-PCR and RDT. The NP samples were collected from patients admitted to the emergency department and from the testing center of the CHU Liège.

Phase 1: Evaluation of samples based on the predefined Ct value. The sample selection took place from 22 October to 27 October 2020. The average SARS-CoV-2 prevalence at the CHU of Liège during the sample selection period was 43.7%. Following the RT-PCR analysis, 15 negative samples and 48 positive samples were selected based on convenience. Positive samples were chosen on the basis of Ct calculated for the N gene by Qiagen RT-PCR in triplicate for each Ct value ranging from 20 to 35. 

Phase 1 bis: Evaluation of samples based on the predefined Ct value after methodology improvement. In order to improve the HeRDT sensitivity, 30 RT-PCR-positive samples were tested according to an adapted protocol between 28 October and 30 October 2020.

Phase 2: Evaluation of randomized samples. The validation was performed from 3 November to 11 November 2020. At the CHU Liège, the prevalence of SARS-CoV-2 positivity during this period averaged 29%. Following the Qiagen RT-PCR analysis, 50 negative and 50 positive samples were randomly selected.

### 2.3. Diagnostic Procedures

RT-PCR: The reference method used was the QIAprep&amp Viral RNA UM Kit (Qiagen, Hilden, Germany), combining liquid-based sample preparation with the one-step RT-qPCR detection of SARS-CoV-2 targets from human respiratory samples. Three different-colored channels (FAM™, HEX™, and Cy5) were used for the multiplex detection of SARS-CoV-2 (N1/N2 genes), human genetic material integrity (B2M and Rnase P), and inhibition control (synthetic transcript), respectively. RT-PCR detection was performed on a LC480 thermocycler (Roche, Basel, Switzerland) according to the following cycling conditions: 50 °C for 10 min, 95 °C for 2 min, 40 cycles at 95 °C for 5 s, 58 °C for 30 s. The quantification of the viral load was carried out using the calibrated standards provided by the Belgian National Reference Centre for Respiratory Viruses so that the virus concentrations could be estimated from the Qiagen Ct values (Table 1). Samples were considered negative when N1/N2 genes were not detected or they presented a Ct value >40.

RDTs: We evaluated the COVID-19 Ag Respi-Strip kit from Coris Bioconcept (CoRDT) and the coronavirus antigen rapid test cassette from Healgen Scientific, LLC (HeRDT). Both assays are qualitative membrane-based immunochromatographic tests that use monoclonal antibodies to detect nucleocapsid proteins from SARS-CoV-2 in direct nasopharyngeal swabs. For both tests, a nasopharyngeal specimen was taken using a sterile swab and the test was performed directly.

HeRDT manufacturer test procedure: The swab was dipped for 1 min in a dropper bottle containing 10 drops of buffer. After removing the swab from the bottle, 4 drops of the solution were placed in the well of the immunochromatographic test. The result was read 15 min later. 

CoRDT manufacturer test procedure: The swab was dipped into a tube containing 8 drops of buffer and stirred thoroughly. After removing the swab, the immunochromatographic strip was placed in the tube. The result was read 30 min later.

Sample processing methods were modified from the manufacturer’s instructions in order to perform the RDT from the same transport media as were used for the RT-PCR. We analyzed the transport medium containing the swab rather than the swab directly, as recommended by the manufacturer. As the tests were carried out in our laboratory, samples arrived in a virus stabilization tube containing phosphate-buffered saline solution. For both tests, 100 μL of transport fluid was added to the buffer.

In addition to the adapted sample use, we evaluated a second modification to the operating protocol to improve the sensitivity (by halving the lysis buffer) on thirty RT-PCR-positive samples. Instead of adding 10 drops of buffer, we added 5 drops for HeRDT and 4 for CoRDT instead of 8. 

The results were read double blind and the technicians performing the RDT were also blinded to the RT-PCR results. 

### 2.4. Clinical Data

We reviewed medical records to find clinical data regarding the presence of COVID-19 symptoms and, when present, the time from the onset of symptoms. 

### 2.5. Statistics

The performance of both CoRDT and HeRDT was evaluated according to the criteria of sensitivity and specificity with a corresponding 95% confidence interval (95% CI). Qiagen RT-PCR was considered as the gold standard for this evaluation. Sensitivity was calculated for two groups: (1) on positive NP swabs including all Ct values and (2) in NP swabs with RT-PCR cycle threshold (Ct) values ≤31. This cut-off was used in the host laboratory to assist with the interpretation of Ct: a positive sample with a Ct value >31, equivalent to a viral load ≤1000 copies/mL, was considered to be a weakly positive result [8,9,15]. The agreement between the two techniques was evaluated using Cohen’s kappa coefficient, κ, with a corresponding 95% CI. Analyses were performed using the R software [19]. 

## 3. Results

For the Phase 1 validation, 63 samples were selected from patients presenting at the COVID testing center of CHU Liège. This cohort included 15 negative (23.8%) and 48 positive NP swabs (76.2%). Positive samples presented RT-PCR Ct values between 20.2 and 35.9 Ct (median Ct = 28.3), corresponding to a SARS-CoV-2 viral load of between 1000 and 10,000,000 copies/mL. Of the 48 positive samples, 29 samples were detected as positive with HeRDT; the test was positive for each of the three specimens in triplicate, reaching a Ct value of 28. However, the results were increasingly inconsistent for those above this value. The sample with the lowest viral load detected with HeRDT presented a Ct value of 31.5 (corresponding to a weakly positive result ≤1000 copies/mL). All RT-PCR-negative samples were negative with both HeRDT and CoRDT, resulting in a specificity of 100%. As shown in Table 1, the sensitivity for HeRDT was 60.4% while CoRDT—which detected 18 positive samples out of the 48 RT-PCR-positive NP swabs—presented a sensitivity of 37.5%. Since the samples with positive RT-PCR presenting a Ct value >31 were usually considered to be weakly positive [15], the performance of both HeRDT and CoRDT was calculated for a subgroup of specimens. This subgroup included all negative samples (*n* = 15) and positive samples with a Ct value ≤31 in RT-PCR (*n* = 39). The improved sensitivity (84.9% for HeRDT and 54.5% for CoRDT) observed in this subgroup is also presented in Table 2.

By halving the volume of the lysis buffer (5 drops instead of 10, with 150 µL of sample) for HeRDT, we observed an improved sensitivity (73.3%). Of the 30 Qiagen RT-PCR-positive samples selected for this validation phase (Ct range: 24.1–33.8; median Ct 29.1), 22 samples were positive with HeRDT. The same specimens were also analyzed with CoRDT, which identified only four positive results out of the 30 RT-PCR-positive samples (leading to sensitivity of 13.3%). The results are shown in Table 3.

**Figure 1:**
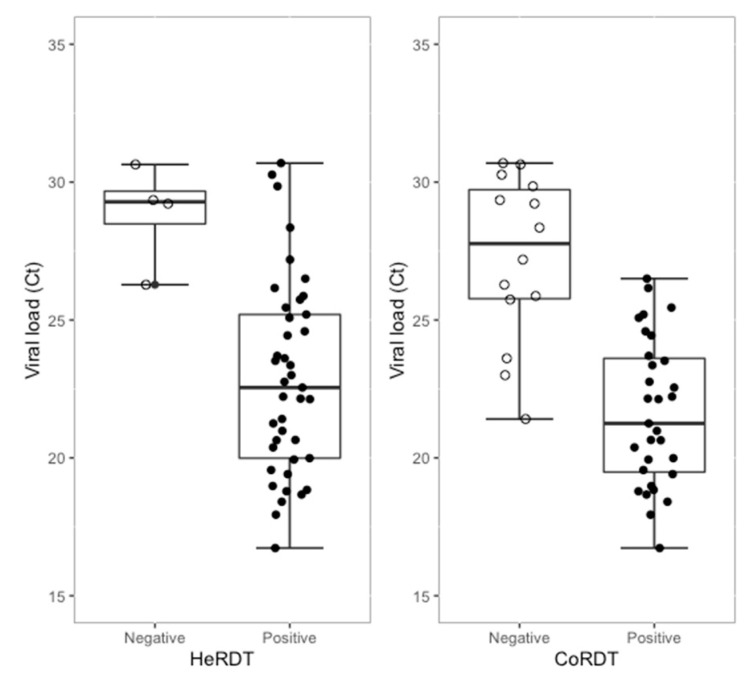
HeRDT and CoRDT results according to the viral load (Ct).

Phase 2 validation was performed on 100 randomly selected routine NP swabs containing 50 negative and 50 positive Qiagen RT-PCR samples, with Ct values in the range of 16.7 to 37.3 (median Ct = 23.6); the viral load ranged from 1000 to >10,000,000 copies/mL) (Figure 1). For NP swabs taken from patients admitted to the emergency department and from the testing center of the CHU Liège, 76% presented COVID-19 symptoms with a median duration of 3 days (from 0 to 11 days), and 86.8% of symptomatic patients had symptoms ≤5 days. As shown in Table 4, HeRDT identified 44/50 positive samples (88% sensitivity). The limit of detection was a sample with a Ct value of 33.8. The κ expressing the agreement between Qiagen RT-PCR and HeRDT was 0.880 (95% CI: 0.788–0.972), which indicated a strong agreement between the two techniques [20]. Thirty-one positive results out of the 50 RT-PCR-positive samples were detected with CoRDT (indicating 62% sensitivity), and the lowest viral load detected had a Ct value of 26.5 (corresponding to a positive result between 1000 and 100,000 RNA copies/mL). The agreement κ index between CoRDT and Qiagen RT-PCR was 0.620 (95% CI: 0.477–0.763), indicating a moderate agreement. All RT-PCR-negative samples were also negative with both HeRDT and CoRDT, leading to a 100% specificity. The performance of both assays was calculated for the subgroup, counting only negative (*n* = 50) and positive samples with a Ct value ≤31 in RT-PCR (*n* = 45). HeRDT showed a sensitivity of 91.1%. Cohen’s kappa coefficient for this subgroup was 0.915 (CI: 0.833–0.997), indicating almost perfect agreement between HeRDT and RT-PCR. In this subgroup, the sensitivity of CoRDT was also improved (68.9%), but the κ expressing agreement between RT-PCR and CoRDT continued to indicate a moderate agreement (κ = 0.699 (CI: 0.560–0.837)).

## 4. Discussion

Effective testing strategies combined with the good performance of diagnostic methods are essential for the control and management of COVID-19 patients, as well as for asymptomatic carriers [21]. Multiple manufacturers have proposed RDTs for the detection of SARS-CoV-2, with reported performances that should be confirmed by clinical validation to reach minimum performance requirements (sensitivity ≥80% and specificity ≥97%) [8]. In this study, we assessed the performance of two RDT kits (CoRDT and HeRDT) in comparison with RT-PCR, which is considered as the gold standard method. The present validation revealed a 100% specificity for both kits, while the sensitivity was, respectively, 88% and 62% for HeRDT and CoRDT. These results were applicable when considering all positive samples from the validation Phase 2. Moreover, we decided to determine the diagnostic accuracy of both RDTs in a subgroup of samples with RT-PCR-positive samples not exceeding 31 Ct. In accordance with the literature and the quantification of the viral load by calibrated standards, a SARS-CoV-2-positive individual with a value >31 Ct (corresponding to a viral load <1000 copies/mL) is considered to be weakly positive [22]. In this studied subgroup, the sensitivity was higher, with 68.9% for CoRDT and 91.1% for HeRDT. In October 2020, Sciensano (National Institute of Public Health in Belgium) updated the COVID-19 testing strategies: RDT can be used as a diagnostic method for COVID-19 in symptomatic patients with symptoms ≤5 days consulting at the emergency department [21]. In fact, antigen detection methods are more effective in patients presenting high SARS-CoV-2 viral loads, most often corresponding to the recent onset of symptoms. As reported in other studies [14,23], a higher sensitivity of RDT was observed when testing patients at an early stage of the disease. Similar results were observed in our study: a predominant subgroup of patients presented COVID-19 symptoms with a symptom onset delay ≤5 days. Clinical data were missing in 24% of samples included in this validation step, while only 8% were taken from asymptomatic patients. Due to the high sensitivity of HeRDT and the updated recommendations from the Belgian authorities for the use of RDTs, we decided to implement HeRDT as a screening test for patients admitted to the emergency department at the CHU Liège. However, all negative antigenic test results had to be confirmed by RT-PCR. 

In addition to the viral load and the number of days of post-symptoms, other factors may influence the performance of the RDT. Among these, our modified sample processing method may impact the results [14,23]. We chose to process RDTs directly from the transport medium, which was also used for RT-PCR, in order to compare results from the same sample. The adaptation led to a dilution of the NP swab that may partly explain the differences in the performance compared to those announced by the kit’s manufacturers or described in other studies [13,24]. Carrying out the RDT directly using the NP swab transport medium—without diluting it in the kit buffer—may improve the sensitivity, as already demonstrated in other studies [14]. 

Rapid diagnostic tests (RDTs) can provide multiple advantages compared to PCR methods. They cost less and are easier to use (simpler equipment, requirement of basic skills compared to molecular tests). They present a shorter turnaround-time compared to RT-PCR and provide results at any hour in the day. However, RDTs may present a series of disadvantages, such as the immunochromatographic tests needing to be read by humans, the interpretation of the results possibly being subjective, and the manual encoding of data being a possible source of error in the Laboratory Information Management System (LIMS), especially in the case of decentralized point-of-care testing configurations. It is essential for laboratories to easily retrieve the results of these RDTs in the LIMS, which can be used for submission to authorities for pandemic monitoring or other purposes. However, the main disadvantage of RDTs is associated with their lower sensitivity compared to RT-PCR, often leading to the verification of negative results [9,25]. 

As previously described [23,24,26], the performance of an RDT depends on the viral load of SARS-CoV-2 in samples, the delay from the onset of symptoms, any adaptation of the testing protocol, and how the results are interpreted by the reader. Moreover, the performance of an RDT is also influenced by the SARS-CoV-2 infection rate; a higher prevalence of infection in the population corresponds to a higher positive predictive value (PPV). Conversely, as the disease prevalence decreases, PPV decreases and the risk of obtaining false positive results increases. During the beginning of the study period (22 October to 27 October 2020), the prevalence of SARS-CoV-2 at the host hospital was 43.7%, leading to a PPV of 100% and a negative predictive value (NPV) of 91.5% for HeRDT. For CoRDT, the calculated predictive values for the same period were 100% for PPV and 77.2% for NPV. For instance, in another context where the prevalence of COVID-19 is 5.0% (prevalence at CHU Liège in January 2021), HeRDT would show a PPV of 100% and an NPV of 99.4%. For CoRDT in the same situation, PPV and NPV would be 100% and 98%, respectively. Contrary to other studies [24], our specificity reached 100%; therefore, it was not possible to predict a PPV. It would be interesting to re-evaluate the specificity during a period with a lower prevalence of COVID-19. It should also be noted that the Phase 2 validation performed in our laboratory was carried out on only 100 samples (50 RT-PCR-positive and 50 RT-PCR-negative NP swabs). However, the European Centre for Disease Prevention and Control (ECDC) recommends testing at least 100 positive and 100 negative samples [25]. At the beginning of this validation study, these recommendations were not yet available. However, a routine daily verification was performed for several weeks and confirmed the sensitivity observed during the second phase of the validation of HeRDT. From 11 November to 31 December, 984 samples (971 negative and 13 positive) were analyzed in parallel using a HeRDT test and QIAGEN RT-PCR assay, revealing a 97% concordance between the two methods. 

Increasing the number of sample tests and the examination of different prevalence conditions remain relevant for validating RDTs and precisely confirming the manufacturer’s diagnostic accuracy in real-life conditions. 

In conclusion, RDTs have a real role in the COVID-19 testing strategy thanks to their ease of use and capacity for mass screening and the rapid detection of SARS-CoV-2 24/7. This validation study showed that it is necessary to confirm the test performances announced by manufacturers before implementing RDTs, especially in the case of protocol adaptation. Although it is crucial to realize that RDT sensitivity is lower than RT-PCR, their use can be of benefit in cases of limited access to molecular methods or when the RT-PCR testing capacity is overburdened.

## Figures and Tables

**Table 1:** Correlation between Qiagen PCR Ct and standard viral quantification.

Interpretation	Viral Load (RNA Copies/mL)	Ct
Very strong positive	>10,000,000	<18.5
Very positive	100,000 to 10,000,000	18.5 to 25
Moderately positive	1000 to 100,000	25 to 31
Weakly positive	<1000	>31

Ct, cycle threshold.

**Table 2:** Phase 1 performance of HeRDT and CoRDT compared to RT-PCR (P = positive, N = negative). The results for all Ct values ranging from 20.2 to 35.9 are presented in (**A**), and only Ct values ≤31.00 are presented in (**B**).

**A.**		**RT-PCR**		**Sensitivity (%)** **(95% CI)**	**Specificity** **(%)**	**Lowest Viral Load (Ct)**
		**P (*n* = 48)**	**N (*n* = 15)**			
HeRDT	P	29	0	60.4 (45.3–73.9)	100	31.51
	N	19	15			
CoRDT	P	18	0	37.5 (24.3–52.7)	100	27.16
	N	30	15			
**B.**		**RT-PCR**		**Sensitivity (%)** **(95% CI)**	**Specificity** **(%)**	**Lowest Viral Load (Ct)**
		**P (*n* = 33)**	**N (*n* = 15)**			
HeRDT	P	22	0	84.9 (68.1–94.9)	100	31.51
	N	8	15			
CoRDT	P	18	0	54.5 (36.4–71.9)	100	27.16
	N	15	15			

CI, confidence interval; CoRDT, COVID-19 Ag Respi-Strip from Coris Bioconcept; HeRDT, coronavirus antigen rapid test cassette from Healgen Scientific.

**Table 3:** Sensitivity of HeRDT and CoRDT compared to RT-PCR after the improvement of operating protocol (P = positive, N = negative). The results for all Ct values ranging from 24.1 to 33.8 are presented in (**A**).

A.		RT-PCR		Sensitivity (%) (95% CI)
		P (*n* = 30)	N (*n* = 0)	
HeRDT	P	22	/	73.3 (53.8–87.0)
	N	8	/	
CoRDT	P	4	/	13.3 (4.4–31.6)
	N	26	/	

**Table 4:** Phase 2 performance of HeRDT and CoRDT compared to RT-PCR (P = positive, N = negative). The results for all Ct values ranging from 16.7 to 37.3 are presented in (**A**), and only Ct values ≤31.00 are presented in (**B**).

**A.**		**RT-PCR**		**Sensitivity (%)** **(95% CI)**	**Specificity** **(%)**	**Lowest Viral Load (Ct)**
		**P (*n* = 50)**	**N (*n* = 50)**			
HeRDT	P	44	0	88.0 (75.0–95.0)	100	33.77
	N	6	50			
CoRDT	P	31	0	62.0 (47.2–75.0)	100	26.5
	N	19	50			
**B.**		**RT-PCR**		**Sensitivity (%)** **(95% CI)**	**Specificity** **(%)**	**Lowest Viral Load (Ct)**
		**P (*n* = 45)**	**N (*n* = 50)**			
HeRDT	P	41	0	91.1 (78.8–97.5)	100	32.84
	N	4	50			
CoRDT	P	31	0	68.9 (53.4–81.8)	100	26.5
	N	14	50			

## Data Availability

The data presented in this study are available on request from the corresponding author. The data are not publicly available due to privacy and ethical concerns.
